# Climate Change, Racism, and Food Insecurity: Cyclical Impacts of Stressors Exacerbate Health Disparities

**DOI:** 10.1007/s40615-024-02202-x

**Published:** 2024-10-16

**Authors:** Christina Ek, James R. Hébert, Daniela B. Friedman, Dwayne E. Porter

**Affiliations:** 1 Department of Environmental Health Sciences, Arnold School of Public Health, University of South Carolina, Columbia, SC USA; 2 Department of Epidemiology and Biostatistics and Cancer Prevention and Control Program, University of South Carolina, Columbia, SC USA; 3 Department of Health Promotion, Education, and Behavior, Arnold School of Public Health, University of South Carolina, Columbia, SC USA

**Keywords:** Climate change, Racism, Dietary Inflammatory Index, Allostatic load, Chronic disease, Health disparities

## Abstract

**Introduction:**

Disadvantaged populations have higher rates of chronic disease, including heart disease, cancer, and diabetes. Race, ethnicity, lower socioeconomic status, and poverty all contribute to these disproportionate rates. Other factors, including systemic racism, climate change, poor diet, lack of food access, and epigenetic influences, that are distributed and experienced differently across vulnerable populations also play a significant role in the development of chronic diseases. This comprehensive review of contributors to chronic diseases emphasizes a unique focus on these identified emerging factors.

**Methods:**

An ad hoc literature review using OVID Medline and Web of Science was conducted.

**Results:**

Findings from prior studies indicate that multiple stressors, both in isolation and in combination, and their negative impacts on both physical and mental health of minorities are exacerbated by climate change.

**Discussion:**

Various stressors dramatically increase chronic disease risk in minority groups. Recommendations for future research to elucidate the impacts of climatic, racial, and dietary adversity with minority populations are presented. Further study in this area is critical for achieving the UN Sustainable Development Goals and improving public health outcomes.

## Introduction

### History of Disease

Historically, acute infectious diseases were major causes of mortality worldwide [1]. However, primarily with the advent of improved sanitation in the late nineteenth century [1–3] and early twentieth century, and secondarily with initiation of large-scale population-based vaccination programs in the 1930s [4], chronic diseases such as heart disease and cancer emerged as more common causes of death and disability [1]. Major chronic diseases encompass those arising from metabolic dysregulation, including Type II diabetes, metabolic syndrome, and autoimmune diseases [5]. Metabolic syndrome results from a combination of factors that increase the risk for diabetes, including high blood glucose, high LDL cholesterol (dyslipidemia), and hypertension. Autoimmune diseases include lupus, Sjogren’s syndrome, rheumatoid arthritis, multiple sclerosis, and Type I diabetes [6]. Chronic diseases are increasing in prevalence worldwide across diverse populations.

Metabolic syndrome and other chronic diseases associated with metabolic dysregulation have become much more widespread due, in large part, to dramatic increases in obesity rates. Chronic diseases are especially common in industrialized nations due to poor diet and sedentary lifestyles and rates are much higher among people of low socioeconomic status (SES) who typically have lower educational attainment [7]. Chronic disease rates are also increasing in low- and middle-income countries (LMICs) as lifestyles become more like those of industrialized nations [8]. Social determinants of health (SDoH), including socioeconomic status and health care access, have major impacts on the prevalence of chronic diseases among diverse populations. Disparities in health care increase the likelihood that chronic diseases are not detected early, and prognoses are worse. Systemic racism results in high-risk minorities living in communities where access to healthy, anti-inflammatory food and safe places to be physically active are lacking [9]. These communities are also more prone to the effects of climate change [9].

In the United States, chronic disease rates are often much higher in disadvantaged racial and ethnic groups, such as African Americans, Hispanics, and Native Americans [8]. These groups tend to have much higher poverty rates, and low SES increases exposure to known risks and hampers access to adequate health care. Furthermore, minority groups may suffer from what has been called “status syndrome,” in which low social standing can have negative psychological consequences that impact health, even after adjusting for classical risk factors, including race, diet, and physical activity [10]. So, even more affluent members of disadvantaged populations can have poorer psychological and physical health due to systemic racism and neighborhood-level environmental factors that can impact quality of life. Low-status individuals feel that they do not have control over their lives, including the wherewithal to engage in healthy behaviors and access to good food and adequate health care [10], and are less likely to engage in environmental activism [11]. Ironically, such activism aims to combat the environmental problems, including climate change, to which they are more vulnerable than higher SES individuals [11]. Research indicates that more vulnerable populations are often highly aware of their heightened risks from climate change [12].

Systemic racism also impedes chronic disease treatment, dramatically increasing risks of complications and poor access to treatment [9]. Furthermore, adverse events and experiences throughout life may make these groups more susceptible to intergenerational effects, which increase future generations’ risk of getting chronic disease [11]. Causes of adverse events include racial discrimination, climate change, and pro-inflammatory diets. For example, Black women are much more likely than White women to have adverse birth outcomes, including preterm or low-birth-weight infants, even after accounting for SES [13, 14]. Both air pollution and heat exposure have been associated with adverse birth outcomes [13]. African Americans have been observed to put much effort into coping with major stressors, a condition known as John Henryism; these efforts negatively impact their collective health [15, 16]. Chronically stressed groups also are more likely to engage in unhealthy behaviors to cope with stress [17].

Racism, climate change, and poor diet hamper efforts to achieve the 17 Sustainable Development Goals (SDGs) proposed by the United Nations [18]. The SDGs are a set of ambitious goals to ensure peace, prosperity, the end of poverty, and optimal health. The goals include zero hunger, gender equality, and resilient communities; stressors threaten all of these aspirations [11].

The Barker Hypothesis has been proposed to explain why some groups have higher rates of chronic metabolic disease [19]. Also known as the developmental origins of health and disease hypothesis, the theory states that poor prenatal nutrition increases the risk for chronic metabolic disease. Poor maternal nutrition alters metabolic signaling pathways [18, 19]. Furthermore, effects can be transmitted through epigenetic mechanisms, in which gene expression occurs without alteration of DNA [20]. The following section describes in greater depth the factors of racism, climate change, and diet and food access contributing to chronic disease disparities. Then, a review of the literature concerning the interrelationships among these stressors and their impacts on disadvantaged populations is discussed. Due to transgenerational effects, adverse impacts of stressors on children and adolescents are described separately from consequences on adults.

## Focusing on Racism, Climate Change, Poor Diet, and Food Access

Major sources of adversity in both LMICs and minority populations in industrialized nations include racism, stressors related to social class and immobility, climate change, and poor diets that promote inflammation [11]. Racism in health care access hinders treatment for many minority groups, regardless of income level. Hate crimes towards several minorities have become more aggressive recently. For instance, hate speech and violence towards Asian Americans both increased with the spread of COVID-19, as the disease allegedly originated in a Chinese laboratory [21]. Hate crimes and racism are major sources of chronic stress for minorities [11, 21].

### Systemic Racism

Stress brought on by systemic racism and other stressors can suppress the immune system, especially cell-mediated immune response, thus resulting in chronic inflammation and associated diseases, including cardiovascular disease [21]. Inflammation is a major underlying substrate for most chronic diseases, including heart disease, cancer, diabetes, and metabolic syndrome. Several signaling molecules are involved in inflammation, including cytokines, C-reactive protein, and TNF-α (tumor necrosis factor-alpha). Cytokine storms occur when the body releases excessive amounts of cytokines, triggering a cascade of other inflammatory responses. C-reactive protein is one of the most frequently measured biomarkers of inflammation [22]. Interleukins are a particular subset of cytokines that regulate intercellular messaging and cellular behavior, including inflammatory responses. Some, like IL-6, which elicits C-reactive protein, are typically pro-inflammatory, whereas others, including IL-10, are typically anti-inflammatory [23]. TNF-α is also usually pro-inflammatory. Factors that promote acute inflammation also tend to suppress chronic, systemic, low-grade, and tissue-specific inflammation. These factors include physical activity and beneficial dietary components, such as capsaicin in chili peppers and many polyphenolic compounds in plants [24]. Poor diets of low-income populations frequently consist of ultra-processed foods, lacking in anti-inflammatory factors [25, 26].

Allostatic load and weathering [27] both refer to the cumulative effect of constant stress exposure and physiological stress reactions, especially frequent triggering of the fight-or-flight response. Disadvantaged groups, due in large part to systemic racism, experience significant allostatic loads and hence weathering. They are worn down physically, mentally, and emotionally by constant stress. Much research has been done on African Americans, but other groups that experience unrelenting racism, such as Hispanics, Asian Americans, and Native Americans, also experience high allostatic loads. High allostatic loads suppress the immune system and promote inflammation [27].

### Climate Change

Climate change disproportionately impacts low- and middle-income countries (LMICs) as well as minority groups in industrialized nations [28]. Declines in crop yields are more severe for farmers in LMICs [28]. Such trends are particularly concerning given that agriculture comprises a much larger percentage of the gross domestic products of LMICs than that of industrialized nations. Different ethnic groups may also be more vulnerable to the effects of climate change than others [29]. Furthermore, in industrialized nations, minorities are more likely to suffer health effects from air pollution. Older adults are especially susceptible; they suffer from higher rates of pulmonary fibrosis and airborne infections such as COVID-19 because of exposure to air pollutants [30]. Children are also particularly vulnerable to the effects of climate change [31]. Production of reactive oxygen species (ROS) from sources ranging from products of combustion, severe air pollution, and pro-inflammatory diets to ionizing radiation is a major contributor to disordered inflammatory responses that result in numerous health problems [32, 33].

The low-income neighborhoods in which minority groups tend to reside are more likely to have fewer trees and other amenities that mitigate air pollutants and moderate high temperatures [34]. Modern housing tends to be more energy-efficient with respect to temperature control. Trees reduce the level of greenhouse gas emissions that contribute to climate change. Discriminatory housing, financing, and redistricting policies, including redlining, have resulted in predominately minority neighborhoods being disproportionately located in low-income, resource-poor areas [34]. These low-income neighborhoods tend to be located near poor educational facilities, as real estate taxes pay for most public primary and secondary education in the United States [34]. These neighborhoods also are more likely to be in the vicinity of facilities that pollute air, such as factories and power plants. Low property values often stem from the presence of these noxious facilities. Children are especially vulnerable to the effects of toxins in poor housing, especially combined with the detrimental impacts of food insecurity. Children in less affluent households, for example, are more likely to have high blood lead levels [31]. Minerals in food compete with environmental lead (e.g., from pipes and paint) for binding sites to prevent absorption into the bloodstream. Therefore, low-SES children in substandard housing are at especially high risk for high blood lead levels. Housing policies combined with climate change are major stressors for low-income minority populations [33, 34].

### Diet

A large portion of the American diet (> 50%) consists of ultra-processed foods, which are extremely pro-inflammatory [35]. Due, in part, to agricultural subsidies on corn and soybeans, these processed foods are much less expensive than fresh items. In several LMICs, ultra-processed foods are commonly purchased because many residents believe that such food is safer to eat than fresh items due to lack of sanitation and refrigeration. The Dietary Inflammatory Index (DII®) quantifies the inflammatory potential of diets and foods; positive values indicate the diet or food is pro-inflammatory. Examples of pro-inflammatory foods are red meat, sugar, confections, and refined grains. Negative index scores indicate anti-inflammatory diets, which tend to have high concentrations of polyphenols and antioxidants from sources including fruits, vegetables, and whole grains. Traditional diets consumed by many cultures at risk for metabolic obesity are anti-inflammatory and reduce risk of chronic disease. They are higher in fiber and micronutrients. In the 1960s, surgeon Denis Burkitt studied rates of colon cancer in both native Africans and African Americans. His results indicated a major reason for lower rates of colon cancer in native Africans was high fiber intake [36]. More current research indicates that fiber intake influences the balance of the gut microbiome [37]. Thus, the beneficial impacts of traditional diets have been well established and have the potential to mitigate some negative effects of systemic racism.

Disadvantaged groups are more likely than Whites to experience health risks associated with obesity, even if their BMI [body mass index = weight (kg)/height (m)^2^] may be < 30 kg/m^2^, the minimum cutoff point at which people are considered obese. Even with concomitant small increases in BMI and adiposity, their risk for chronic disease dramatically increases [38, 39]. They are therefore considered to be “metabolically obese” [40]. The “thrifty gene” hypothesis posits that these groups are more likely to be metabolically obese because their societies were traditionally prone to famine. As a result, they inherited genes that enabled them to gain excessive body fat during times of abundant food supply to shield against famine [38]. Such genes are now disadvantageous when calorically dense food is generally abundant, even though food insecurity, which includes many factors beyond caloric intake, is still a major issue worldwide. Furthermore, many of these cultures view heavyset people as attractive or at least higher status or more affluent. This mindset contrasts with American culture, which tends to value thinness to a great extent, especially in females. Such derision of overweight individuals ignores the influence of food industry corporations and government officials who support policies, including agricultural subsidies, that render ultra-processed foods inexpensive. Although the focus of research tends to be on noncommunicable chronic diseases, the risks of infectious diseases, such as COVID-19, can also increase with obesity [41].

### Food Insecurity and Food Access

The four dimensions of food security are access, availability, utilization, and stability [42]. Access refers to the ability of individuals to obtain food through adequate resources. Availability refers to the food supplies available to individuals. Utilization refers to the proper storage and use of food, including allocation in households. Stability refers to access, availability, and utilization being available consistently and on a regular basis. Food yields worldwide, particularly of cereals, are sufficient to provide for the entire global population. However, issues related to distribution and access limit the ability of all individuals to achieve all four dimensions of food security, particularly in conflict-ridden LMICs.

A major component of food insecurity worldwide is micronutrient deficiencies [43, 44]. Micronutrients are required only in very small amounts, several orders of magnitude lower than the macronutrients, including carbohydrates, proteins, or fats. The impacts of micronutrient deficiencies are not as visible as those of insufficient caloric intake, yet still are profound; they are therefore referred to as “hidden hunger.” Deficiencies of iron, vitamin A, and iodine are common worldwide, especially in LMICs. Effects of micronutrient deficiencies are wide-ranging, affecting many aspects of metabolism. However, in general, these deficiencies tend to lower productivity and lead to much disability. They lower quality of life, as indicated by disability-adjusted life years (DALYs), a measurement of how many years disabled or ill individuals lose due to their conditions. Iron-deficiency anemia lowers workplace and school productivity, and iodine deficiency (goiter) is a major cause of cognitive impairment worldwide [44]. Black women in the United States have higher maternal mortality rates, due to both systemic racism and iron-deficiency anemia. Women in LMICs, especially in sub-Saharan Africa, also have higher maternal mortality rates due, in part, to high incidence of iron-deficiency anemia [44]. Vitamin A deficiency results in progressively poor vision and eventual blindness, and can also exacerbate measles, a common and potentially fatal infectious disease in LMICs. Micronutrient deficiencies have severe negative impacts worldwide.

The double burden of malnutrition is particularly common in LMICs that are undergoing a “nutrition transition,” in which diets are becoming more like those of industrialized nations, i.e., with large amounts of ultra-processed foods [45]. The double burden refers to overweight adults and underfed children in the same household [46, 47]. Frequently in these households, women are thin and children’s growth is stunted [46]. Increased consumption of ultra-processed foods negatively impacts diets, especially as residents of LMICs shift away from their traditional anti-inflammatory diets to much more pro-inflammatory diets. Diets in urban areas of LMICs are becoming more like those of the poor in industrialized nations; that is, their diets are increasingly more pro-inflammatory [45]. Ironically, diets of wealthy residents of industrialized nations tend to be similar to those of the poor in LMICs, and, as such, are much more anti-inflammatory. However, various LMICs are in different stages of the nutrition transition. Many countries are in stage 4, a major cause of chronic disease. Disadvantaged groups in industrialized nations are frequently at earlier stages compared to more affluent groups [47].

Ethnic and racial minorities are much more likely than Whites to experience psychological stress related to racism and climate change, and their diets are more likely to be pro-inflammatory. Pro-inflammatory diets, along with stressors such as climate change and racism, suppress their immune systems and make them more vulnerable to health problems, particularly chronic diseases such as heart disease and diabetes.

## Methods

### Ad Hoc Literature Review

A literature review was conducted with the objective of determining the breadth of knowledge concerning the synergistic impact of multiple stressors on disadvantaged populations and to determine shortcomings in prior research. Articles with the terms “climate change,” “Black or African American,” “Hispanic,” “Alaska Native or Native American,” “Asian or Asian-American,” “adverse childhood experiences,” “racism,” “epigenetics,” and “metabolic syndrome” were obtained from OVID Medline, a collection of research published in medical journals and indexed by the National Library of Medicine (NLM) via Medline. Other terms searched included “allostatic load,” “weathering,” “inflammation,” “environmental justice,” “diabetes,” and “redlining.” These terms were also combined by using the “and” function on OVID Medline. The “or” function was also used, as in “Black or African American” and “(climate change or racism).” Other search term combinations included “climate change and adverse childhood experiences,” “epigenetics and Black or African American,” “allostatic load and weathering and Black or African American,” and “racism and epigenetics metabolic syndrome.” Web of Science was also searched using these same terms. Over one hundred abstracts were scrutinized for salience, and papers selected sufficiently discussed stressors either in isolation or in combination. Many manuscripts were rejected because they were literature reviews. Furthermore, many of the climate change articles were rejected, as they discussed impacts on animals or wildlife rather than humans. Limits were not placed on publication dates. The following sections describe studies obtained from the ad hoc literature search. Studies conducted on either children or adolescents will be discussed, followed by studies that involved adults.

## Results

### Impacts of Stressors on Children and Adolescents

Children in disadvantaged groups are more likely to experience adverse childhood experiences (ACEs) more frequently than White children. These ACEs include abuse (physical or emotional), parental incarceration, observing spousal abuse, and food insecurity [48]. Climate change is also a major source of ACEs [49]. ACEs are not mutually exclusive; multiple types frequently occur simultaneously. Childhood malnutrition impedes physical, social, and mental development. For example, stunted growth presents in the double burden increases the risks for debilitating obesity and chronic diseases [46, 47]. Malnourished children also perform poorly in school [50]. Chronic diseases have become more common in children along with obesity. Type II diabetes previously was also known as “adult-onset diabetes,” as it was heretofore unknown in younger populations. However, Type II diabetes rates have dramatically increased in children concomitantly with obesity rates. High obesity rates have also increased the risk for nonalcoholic fatty liver disease (NAFLD), now also known as metabolic dysregulation–associated steatotic liver disease (MASLD) [51]. Adolescents experience many of the same issues as children. However, the effects are not as profound due to their later stage of development. Nonetheless, adversity and high stress levels impact their cardiovascular health and lead to metabolic dysfunction.

Ducharme-Smith et al. [52] conducted a cross-sectional study on NHANES data from between 2005 and 2016. They determined that African American adolescents for whom diets were more anti-inflammatory had lower risks of hypertension and other components of metabolic syndrome. However, the cross-sectional nature of the study limits the ability to make inferences about temporal relationships. Furthermore, the use of even a single 24HR recall as the dietary assessment in NHANES data presents fewer disadvantages than food frequency questionnaires (FFQs), which are typically used in large-scale epidemiologic studies. Although the NHANES typically uses only one 24HR recall, for some individuals, two recalls are administered resulting in less bias. FFQs are not as precise as 24HR recalls in assessing foods consumed, as they require participants to recall food intake over a much longer time period, typically a year. Furthermore, they do not include all possible foods consumed, especially ethnic foods or dishes. However, 24HR recalls are expensive to conduct and require extensive interviewer training. Furthermore, a prospective cohort study conducted by Matthews et al. [53] followed African American boys from childhood to adulthood, approximately 30 years of age. Those with depressive symptoms in childhood were more likely to smoke. Although depressive symptoms were not statistically associated with inflammation or metabolic syndrome, smoking tobacco is associated with higher rates of most chronic diseases, especially cancer and cardiovascular disease. Tobacco is, in and of itself, a strongly pro-inflammatory agent, thereby increasing chronic disease risk [54].

Other studies on children and adolescents focused on other minorities. Priest et al. [55] studied ACEs in indigenous Australian children, especially racial discrimination, and impacts on metabolic markers, including inflammatory responses, BMI, and blood pressure. Indigenous children who had been exposed to at least two episodes of racial discrimination had significantly higher levels of inflammatory markers, as well as BMI, blood pressure, and waist circumference. Similarly, Burns et al. [56] studied American Indian and Alaskan native children and determined several factors that lead to poor health outcomes, including increased rates of diabetes and obesity. These factors, including air pollution and low cost of ultra-processed foods, were associated with government-enforced relocation and historical trauma due to racial discrimination.

## Impacts of Stressors on Adults

Research on adults was much more extensive. Adversity throughout childhood and adolescence can also impact adult health, as well as that of future generations. Rates of chronic diseases generally increase with age, although children can develop them as well. Epigenetic effects can be expressed across generations even if the nucleotide sequences of DNA are unaffected. Therefore, trauma can increase the risk of disease in future generations through epigenetic changes [19], especially DNA methylation, in which methyl groups, which include three hydrogen atoms bonded to a carbon atom, are added to DNA. Methylation is the most studied of all epigenetic mechanisms. Other mechanisms include histone acetylation. Epigenome-wide association studies (EWAS) examine associations between DNA changes and risks of disease, including cancer and autoimmune diseases. For example, Chitrala et al. [57] determined multiple DNA-methylated positions and regions that were associated with metabolic syndrome in both Blacks and Whites. Barcelona et al. [58] examined DNA methylation in African American mothers and their children. Although no DNA methylation sites were associated with traumatic experiences in mothers, one site on the ENOX1 gene was associated with trauma in children. The gene is associated with depression and other mental health disorders, rendering children with the gene more susceptible to the impact of ACEs.

Multiple studies indicate that these epigenetic mechanisms continue to have deleterious impacts for several generations. Deep learning describes entrainment in physiological and biological systems. For example, the thymus gland is not functional after adolescence. However, prior to that period, the gland trains bone marrow on how to make T-cells to fight infections [59]. Similarly, deep learning increases the risk for chronic disease in groups exposed to continuously high stress levels. Denis et al. [60] determined cytokine markers associated with Type II diabetes, metabolic syndrome, and obesity. Dawson et al. [61] examined the impact of different forms of both direct and indirect racism on the mental and physical health, including inflammatory markers, on women of various races (White, Black, Hispanic, and Asian-American). Women who experienced more racism-related stress had lower self-esteem and concomitant higher levels of inflammatory markers, including C-reactive protein. Subtle acts of racism known as microagressions were associated with poor mental and physical health. Although the study included many different races, the sample only involved women, of whom almost three-quarters were European-American. Zilioli et al. [16] recruited African American adults, who completed questionnaires concerning childhood SES, levels of John Henryism, and demographic information. The researchers also measured blood pressure and waist circumference as well as triglycerides, hemoglobin, and C-reactive protein levels. They determined that John Henryism was correlated with low childhood socioeconomic status, but not inflammation markers. Ammous et al. [62] studied DNA methylation sites and presence of atherosclerosis in African Americans. They determined associations between atherosclerosis and sites on the AHRR, GF11, and LRC552 genes. However, they only initially recruited 391 African Americans, and high levels of attrition occurred during the study time frame. Creighton et al. [63] determined many DNA methylation sites that were associated with increased risk of prostate cancer.

Studies also examined inflammatory responses among adults in minority groups. Johnson et al. [64] studied C-reactive protein and cortisol (stress hormone) levels in Black women. High levels of C-reactive protein were associated with lifetime stress levels, whereas high levels of cortisol were associated with more recent stress related to racism. Cave et al. [65] studied indigenous Australian adults and impacts of racial discrimination on allostatic load. Results indicated that although indigenous Australians exposed to racial discrimination tended to have higher allostatic loads, whether the differences were statistically significant depended on specific circumstances. For example, both older adults and those of low SES were more likely to have higher allostatic loads.

Impacts of stressors were shown to vary by race and ethnicity, as well as by nation. Peek et al. [27] studied allostatic load in foreign-born Mexicans, Mexican Americans, Blacks, and Whites, in Texas. Foreign-born Mexicans had lower levels of C-reactive protein, IL-6, and TNF-α than Mexican Americans. Mexican Americans and Blacks both had the highest allostatic loads, as determined by these biomarkers. Their participants lived near oil refineries, a characteristic that limits generalizability, but also underlines the importance of environmental justice as it applies to low socioeconomic status. Saelee et al. [66] studied National Health and Nutrition Examination Survey (NHANES) data to determine prevalence ratios for allostatic loads, based on cardiovascular and metabolic markers. Results indicated that both Black men and women in food-insecure households had significantly higher allostatic loads, as did Hispanic women in marginally food-secure households. Shaker et al. [67] examined thousands of census tracts throughout the United States, including degree of redlining and level of food access. Census tracts in areas that had undergone redlining and therefore had greater percentages of Blacks, Hispanics, or other minorities had fewer grocery stores. Results from Elenwo et al. [68] indicated that minorities that experienced more racial discrimination also tend to be more food insecure. Brown et al. [69] found that food insecurity rates differed among college students with respect to race; Black college students were more likely than students of other races or ethnicities to be food insecure, as well as have less social support Although few studies have researched the effects of stressors on populations in LMICs, Selvamani et al. [70] determined levels of food insecurity and perceived stress among older individuals in six LMICs: Ghana, South Africa, Mexico, Russia, China, and India. In India, Russia, China, and Ghana, the associations between levels of food insecurity and perceived stress were statistically significant, but not in Mexico or South Africa. Roberts et al. [71] determined prevalence of cardiovascular disease and risk factors, including hypertension, among Haitian adults. They indicated that Haitians who were more socially vulnerable were more likely to be afflicted with cardiovascular disease or be hypertensive. Social vulnerability included low socioeconomic status, poor housing quality, lack of water treatment methods, and neighborhood violence.

Relatively few studies reviewed focused on climate change, either on its own or combined with other stressors. Experimental designs to elucidate such impacts are more difficult, as climate change will have a multitude of effects that differ worldwide and exposures will vary widely over different geographical locations as well as over time. As noted, LMICs will likely be affected to a much greater extent than industrialized nations. Multiple studies in the ad hoc review indicate that, ironically, populations that consume fewer resources compared to more affluent populations are more likely to bear the brunt of climate change. Nations or communities with few resources are less equipped to withstand the rigors of climate change. For example, low-income neighborhoods are frequently several degrees hotter than more affluent neighborhoods. Low-income neighborhoods have fewer trees and other features that reduce average temperatures [72]. Climate change can lead to ACEs, especially when populations are displaced due to droughts, floods, or other extreme events. Refugees are likely to experience adverse physical and mental health effects from extreme chronic stress [73]. Climate change is also a major factor in political and societal strife, which can lead to conflicts that place further stress on minority, low-income populations [74, 75]. Parochialism, in which members of a group are biased against those of other groups, can lead to conflicts resulting from migration brought about by climate change [73]. Climate change can result in a variety of psychological impacts in minority groups, including heightened risks of depression and posttraumatic stress disorder [74, 75].

**Fig. 1:**
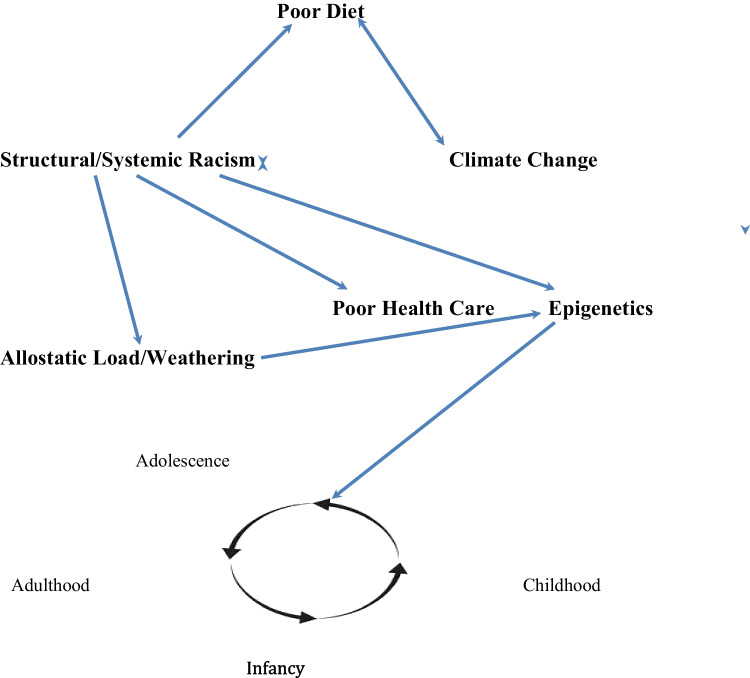
Syndemic representation

Racism, poor diet, food insecurity, and climate change, both in isolation and combination, have severe consequences for minority populations. They produce chronic stress that gradually wears away at people’s ability to remain healthy and free of chronic disease, especially inflammatory conditions. The term “syndemic” has been used in prior research to refer to a constellation of conditions or factors that contribute to each other and occur together in populations, such as these chronic stressors [76, 77]. Table 1 summarizes results of previous research on stressor impacts on disadvantaged minority groups. Figure 1 depicts synergistic interactions among stressors, which disproportionately impact minority populations.

**Table 1:** Adversity research summary

Authors	Year	General findings
Zilioli et al. [16]	2022	John Henryism associated with low childhood socioeconomic status
Peek et al. [27]	2010	Mexican Americans and Blacks had higher pro-inflammatory biomarkers than Caucasians or foreign-born Mexicans
Ducharme-Smith et al. [52]	2021	African American adolescents with higher diet quality had lowered risk of metabolic syndrome
Matthews et al. [53]	2020	Childhood depressive symptoms were associated with smoking, but not with either metabolic syndrome or inflammation
Priest et al. [55]	2020	Allostatic load higher in Australian children exposed to racial discrimination
Burns et al. [56]	2021	Air pollution associated with poor health outcomes in Alaskan native and American Indian children; also historical trauma and relocation negatively impacted health
Chitrala et al. [57]	2020	Specific DNA methylation sites differed in Blacks and Whites with metabolic syndrome
Barcelona et al. [58]	2022	No association of DNA methylation with maternal trauma; site on the ENOX1 gene associated with trauma in children
Denis et al. [60]	2018	Cytokine markers associated with Type II diabetes, metabolic syndrome, and obesity
Dawson et al. [61]	2022	Women exposed to more direct and indirect forms of racism had lower self-esteem and higher levels of inflammatory markers
Ammous et al. [62]	2022	Multiple DNA methylation sites on several genes were associated with higher rates of atherosclerosis in African Americans
Creighton et al. [63]	2023	Multiple DNA methylation sites associated with prostate cancer
Cave et al. [65]	2020	Allostatic load higher in Australian adults exposed to racial discrimination
Saelee et al. [66]	2024	Food-insecure Blacks had higher allostatic loads
Shaker et al. [67]	2023	Redlining associated with poverty and food insecurity
Elenwo et al. [68]	2024	Food insecurity linked to poor health outcomes in children
Brown et al. [69]	2023	Multiple determinants of food insecurity among college students, especially Blacks
Selvamani et al. [70]	2022	Allostatic load higher in food-insecure older adults in many LMICs
Roberts et al. [71]	2024	Haitians from whom social determinants of health were less favorable more likely to be afflicted with cardiovascular disease and hypertension

Table 1 displays synopses of prior research concerning the effects of adversity on minority populations.

Figure 1 vividly illustrates interrelationships and consequences of racism, climate change, and poor diet. Note the two-way arrows. Climate change can worsen food insecurity, but pro-inflammatory diets can also exacerbate climate change. Industrialized meat production is a major source of greenhouse gas (methane) emissions. Racism and climate change can also intensify each other. Although LMICs are projected to bear the brunt of climate change’s impacts, industrialized nations, which have the resources to combat climate change, can be unwilling to assist LMICs, which tend to have predominantly non-Caucasian populations. Climate change also impacts low-income communities to the greatest extent.

## General Discussion

Minority groups in industrialized nations and residents of LMICs are at elevated risk of chronic diseases, including heart disease, cancer, and diabetes. Diseases associated with metabolic dysregulation are also more likely to be severe, with increased risk of complications and poor outcomes, including blindness and kidney disease. These comorbidities also compromised immune function and put individuals afflicted at increased risk of many other conditions, including common infections such as COVID-19 [78, 79]. A multitude of factors contribute to this increase, including racism, climate change, poor diet, and food insecurity. Racist policies keep many members of minority groups mired in poverty. Cumulative stress responses from racism, climate change, and other sources of adversity prime minorities for chronic diseases brought on by inflammation. Epigenetic mechanisms result in adversity impacting future generations. Thus, stressors have continual cyclical impacts on the health of minorities, regardless of socioeconomic status. Results obtained from the studies reviewed above indicate a variety of physical and psychological effects of stressors on minorities.

## Limitations and Future Directions

More research is needed to understand the impacts of these syndemics on underserved and higher-than-average risk populations. Climate change research is particularly lacking, in part because designing studies is challenging. Interactions among stressors need to be analyzed to a greater extent; as noted, they hinder achievement of the SDGs. [80]. Our ad hoc literature review was meant to identify gaps in our understanding of the association between climate change, racism, and food systems. As such, we did not conduct an exhaustive search of the literature to identify, for example, studies on multiple racial and ethnic groups; thus, a systematic literature review was not undertaken. However, searching such a complicated set of relationships would be lengthy.

Our search did indicate that groups other than African Americans need to be emphasized in future research. Research has not been extensively conducted on Native Americans, Hispanics, Asian Americans, and those of mixed ancestry. Most studies surveyed involved African Americans; only a fraction studied other minority groups such as Mexican Americans and Native Americans [27, 56]. Studies could also address whether participants self-identify as members of a particular racial or ethnic group, as racial or ethnic identification could be another source of stress and therefore increased allostatic load. Studies also need to be conducted in other regions, such as in South America, sub-Saharan Africa, and South and Southeast Asia. The overwhelming majority of research has been conducted in the United States. Although racism in the United States is very well documented, racism is prevalent worldwide. In South America, for example, many countries, including Brazil and Colombia, have large *mestizo* (mixed indigenous and European ancestry) populations and other populations of African descent [81]. Therefore, racism directed towards these *mestizos* or Blacks is also pervasive. Slavery has also been prevalent in many other areas, such as between Asia and Africa with the dhow trade over many centuries [82]. Modern-day slavery also cannot be disregarded. To examine these relationships, other search engines could be utilized.

Furthermore, another important issue concerns impacts on inflammation biomarkers or other health outcomes. In addition to cytokines and C-reactive protein, relevant biomarkers need to be more extensively studied. Epigenetic studies could also study mechanisms other than DNA methylation, such as histone acetylation to a greater extent. Much research has been conducted on cardiovascular disease or diabetes. Research on other diseases, especially autoimmune disorders, could be emphasized.

Most research has also been conducted on adults, especially females. Children and adolescents need to be more extensively researched. Studies of many different age groups enhance knowledge about the period of etiologic relevance, when individuals are most susceptible to stressors and the resulting impact on inflammatory responses and chronic diseases. Sex differences need to be studied to a greater extent; for example, women are more likely to be afflicted by autoimmune diseases. It has been theorized that their immune systems are more vigilant to prepare for childbearing and lactation. An overly alert immune system is more likely to attack healthy tissue. Although evidence clearly indicates associations among racial discrimination climate change, and poor diet combined with food insecurity with chronic diseases linked to long-term inflammation, more studies of longer duration are needed. Large sample sizes would need to be emphasized. Although many studies reviewed included over two thousand participants, several involved very small sample sizes: 170, 108, and 34 participants, respectively [16, 61, 64].

## Conclusion

Trauma and chronic stress are associated with high rates of chronic disease, especially in minority populations. Stressors include racism, climate change, and poor diets that promote inflammation. These disparities hamper progress with regard to the UN Sustainable Development Goals. Complex sets of factors combine to produce health disparities, both in industrialized nations and in LMICs. Stressors have cascades of negative health effects that can become cyclical through epigenetic mechanisms. Research on disadvantaged populations, chronic disease, inflammation, epigenetics, and stressors is lacking in many respects. It is striking that, although no constraints were placed on publication date, most research has been conducted since 2010. Multiple prospective cohort studies could be conducted to determine impacts on Hispanics, Asians, Native Americans, and other racial and ethnic groups. Elucidation of the multitude of mechanisms involved could support resilient communities that are sustainable and environmentally just.
